# New Classification of Autologous Tooth-Derived Grafting Materials: Fundamental Concepts

**DOI:** 10.1155/ijod/6646405

**Published:** 2025-07-23

**Authors:** Elio Minetti, Silvio Taschieri, Marco Berardini, Stefano Corbella

**Affiliations:** ^1^Department of Biomedical, Surgical, and Dental Science, University of Milan, Milan, Italy; ^2^IRCCS Orthopedic Institute “Galeazzi”, Milan, Italy; ^3^Department of Oral Surgery, Institute of Dentistry, I. M. Sechenov First Moscow State Medical University, Moscow, Russia; ^4^Private Practitioner, Pescara, Italy

**Keywords:** autogenous tooth, biomaterials, bone regeneration, classification, dentin graft, none substitutes, tooth bone grafting, tooth graft

## Abstract

The use of dentin grafts is relatively recent, and their efficacy remains a topic of debate. Various techniques and devices are available for dentin grafting; however, their application has been inconsistent, as each method yields a distinct product with unique biological properties and potential uses. One of the challenges that arises with the introduction of a new biomaterial is the potential confusion between different preparations. Specifically, generalization may significantly impact the understanding of unique qualities and/or potential limitations. The various materials that make up the family of tooth-derived graft materials share only one common starting point: the patient's extracted tooth. Beyond that, the processes of grinding, demineralization, and detoxification differ significantly, resulting in final materials with completely different percentages of minerals, bone morphogenetic proteins (BMPs), collagenic and noncollagenic proteins, and residual bacterial load. These differences influence the regenerative potential of one material compared to another, as well as the resorption rate. For instance, incomplete sterilization of the material can accelerate the resorption process, leading to insufficient regeneration. Here, we propose a classification of dentin grafts into four categories based on their processing methods. This classification aims to clarify the successes and challenges encountered to date, offering an objective framework to guide the ongoing development of these techniques. The aim of this study is to establish the first classification system of autogenous partial demineralized tooth-derived grafting biomaterials.

## 1. Introduction

Trauma, destructive caries, periodontal disease, osteolytic lesions, and neoplastic conditions are the most common causes of tooth loss, leading to partial or complete edentulism of the maxillary bones. This results in significant social impact and a decrease in quality of life due to impairments in masticatory, phonatory, and esthetic functions [1, 2].

The loss of one or more teeth typically results in three-dimensional bone resorption, leading to changes in occlusal, musculoskeletal, and joint relationships, as well as gradual facial aging. This volumetric bone loss, which can interfere with proper rehabilitation of the maxillary bones, appears to be associated with the loss of the functional masticatory load on the alveolar bone [3]. Some authors demonstrated that following a tooth extraction, alveolar bone resorption can range from 2.5 to 7 mm in height and up to 30 mm in width [4].

Fixed rehabilitations, in these cases, often require bone augmentation procedures to allow standard titanium implants insertion. Over the past decade, numerous studies have assessed the most effective surgical techniques and the ideal grafting materials for the reconstruction of bone defects [5]. Many surgical techniques were suggested with or without the use of graft materials and resorbable or nonresorbable membranes [6, 7].

As to the grafting material, the international literature proposed fresh or demineralized freeze-dried human bone, animal (xenograft), and synthetic (allograft) materials, used either alone or in combination, to augment hard tissue dimensions [8].

For many years, the autogenous bone was regarded as the “gold standard” due to its osteogenic, osteoconductive, and osteoinductive properties. However, it presents certain challenges, including donor site morbidity, surgical complications, limited availability, and, in some cases, a high resorption rate [9].

For these reasons, in the last 10 years, great effort was applied in developing various biomaterials starting from animal bone, with rapid or slow reabsorption, used as scaffolds that show only osteoconductive properties [10, 11]. Therefore, different xenograft processing procedures can affect its resorption rate and its clinical features as well as its osteoconductivity. In fact, chemicals used to eliminate cells and proteins could damage bone constituents, harm osteoinductivity, and change the scaffold structure by increasing the hydroxyapatite (HA) crystal size [12, 13]. For this reason, biomaterials of the same origin could be different from each other because of the various production and antigen processing methods (such as heat or enzymatic treatments), which result in final products with distinct properties and compositions specifically influenced by the production processes [14].

At some point, scientists began to consider the alveolar bone differently, an integral part of the dental tissues, and they noticed that bone and teeth share the same mesenchymal embryonic origin [3]. Numerous studies have shown that teeth and bones have similar chemical compositions, with dentin (*D*) consisting of 70% inorganic material and 20% organic material, compared to alveolar bone, which is made up of 65% inorganic material and 25% organic material. Additionally, both tissues contain type I collagen (COL-I; 90%) and noncollagenous proteins (10%), such as osteocalcin, osteonectin, sialoprotein, and phosphoprotein, which are crucial for bone matrix formation and mineralization. Growth factors are also present, including mineralization protein LIM-1 (LMP-1) and transforming growth factor-β (TGF-β) [15–17].

In addition, in 1967, some Authors discovered the osteoinduction potential features of demineralized dentin matrix [18, 19], and some years later, Bessho et al. were able to detect the presence of bone morphogenetic proteins (BMPs) in a human dentin matrix after a demineralization process [20]. This significant discovery demonstrated that partially demineralized human dentin could be successfully utilized as an osteoinductive grafting material.

In 2017, Rijal theorized how the dentin demineralization process of autologous extracted teeth allows better bone augmentation for the increased availability of BMPs [21]. Kim et al. [22] and Minamizato et al. [23] showed the efficacy of a chairside-prepared autologous partially demineralized dentin matrix for clinical bone regeneration procedures in humans.

Tooth graft material could support and enhance regeneration procedures such as socket preservation, sinus lifts, and horizontal and vertical regeneration [24–28]. An experimental study on rabbits [29], in 2018, demonstrated that particulate human tooth graft created significantly higher new bone formation compared to deproteinized bone.

One of the challenges that arises with the introduction of this autogenous biomaterial is the potential confusion between different preparation methods. Specifically, generalization may significantly impact the understanding of unique qualities and/or potential limitations.

The authors of the present article feel that it is necessary to clarify and classify the novel family of biomaterials derived from autogenous dentin. Although the starting point for all these materials is the same, the patient's extracted tooth, the transformation procedures lead to final grafting materials that can possess different features. The aim of this study is to establish the first classification system for autogenous dentin-derived biomaterials.

## 2. Materials and Methods

Different medical devices and techniques have been developed over the years to transform chairside, the extracted tooth of the patient into a suitable osteoinductive particle graft, but distinct clinical preparation protocols give different results in terms of detoxification and preservation of autogenous collagenic and osteoinductive proteins. It has been performed a literature review by searching the keywords dentin graft, tooth graft, tooth bone grafting, and demineralized dentin to find all the clinical procedures suggested and validated. All the preparation methods are summarized in Table 1.

**Figure 1:**
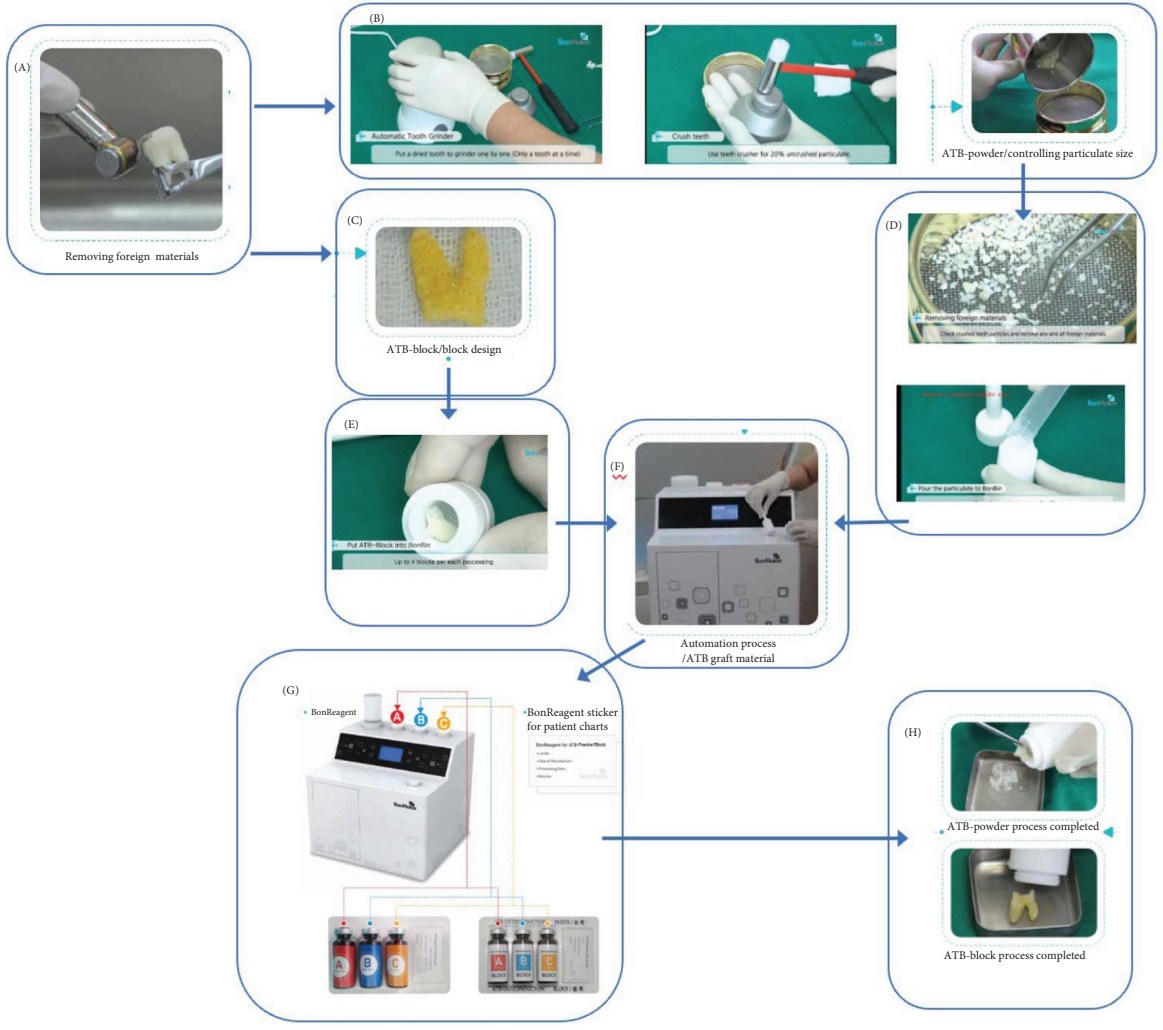
BonMaker visual representation of both block and particles graft preparation protocol (source: YouTube and BonMaker brochure). (A) After tooth extraction, the enamel and cementum of teeth are removed using a high-speed handpiece. (B) High-speed grinder is used to crush the tooth. The hammer is used to crush the pieces too big. The filters are used to sieve dental particles. (C) Tooth prepared to be a block with holes. (D) Dental tissue after the initial grinding and inserted in the container. (E) Dental block inserted in the container. (F) The container is inserted in the device. (G) The three different liquids are inserted inside the device manually. Same procedure with the block with different liquids. (H) Granules and block obtained after the process.

**Figure 2:**
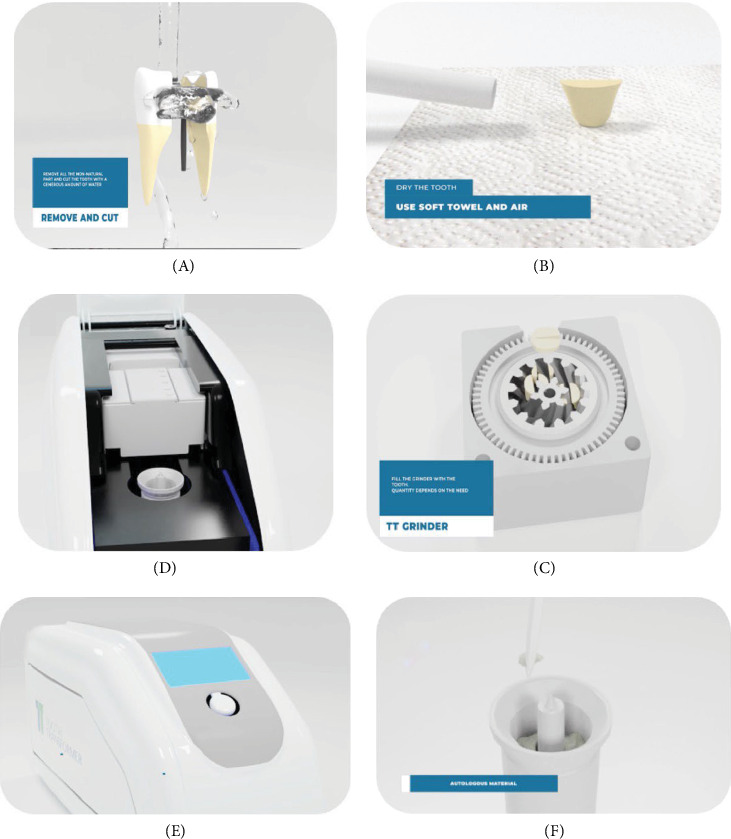
Tooth Transformer visual representation of the preparation protocol (source: Tooth Transformer video presentation). (A) After tooth extraction, the teeth are cleaned from the filling using a high-speed handpiece and sectioned in small pieces. (B) The teeth need to be dry. (C) Insertion of the dry pieces into the low-speed grinder. (D) Insertion of the three components (grinder filled with the teeth pieces, single use cartridge, processed granules container). (E) Push the button to start the procedure. (F) End procedure with processed granules.

**Figure 3:**
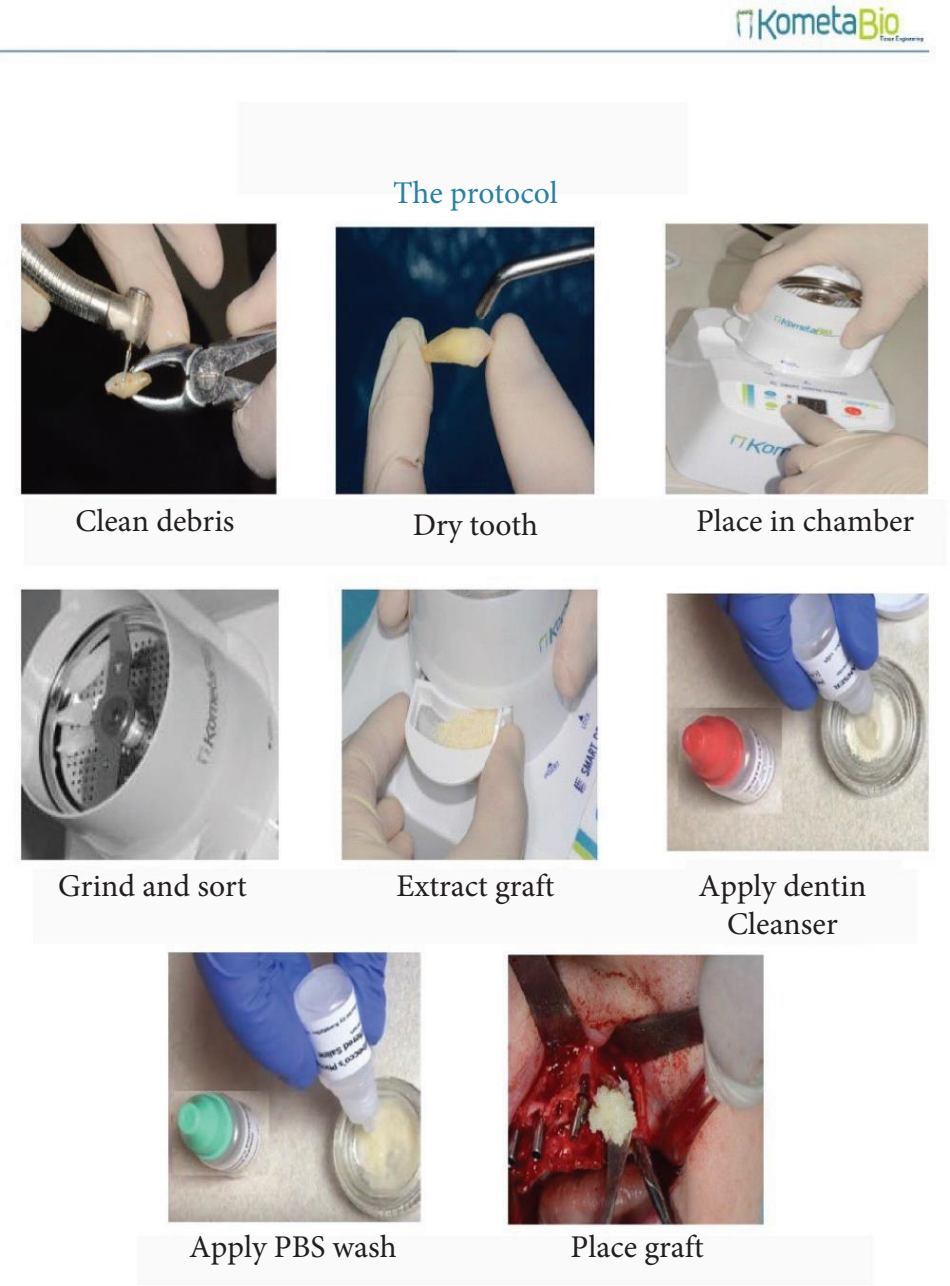
Smart Dentin Grinder visual representation of the preparation protocol (source: Smart Dentin Grinder brochure).

**Figure 4:**
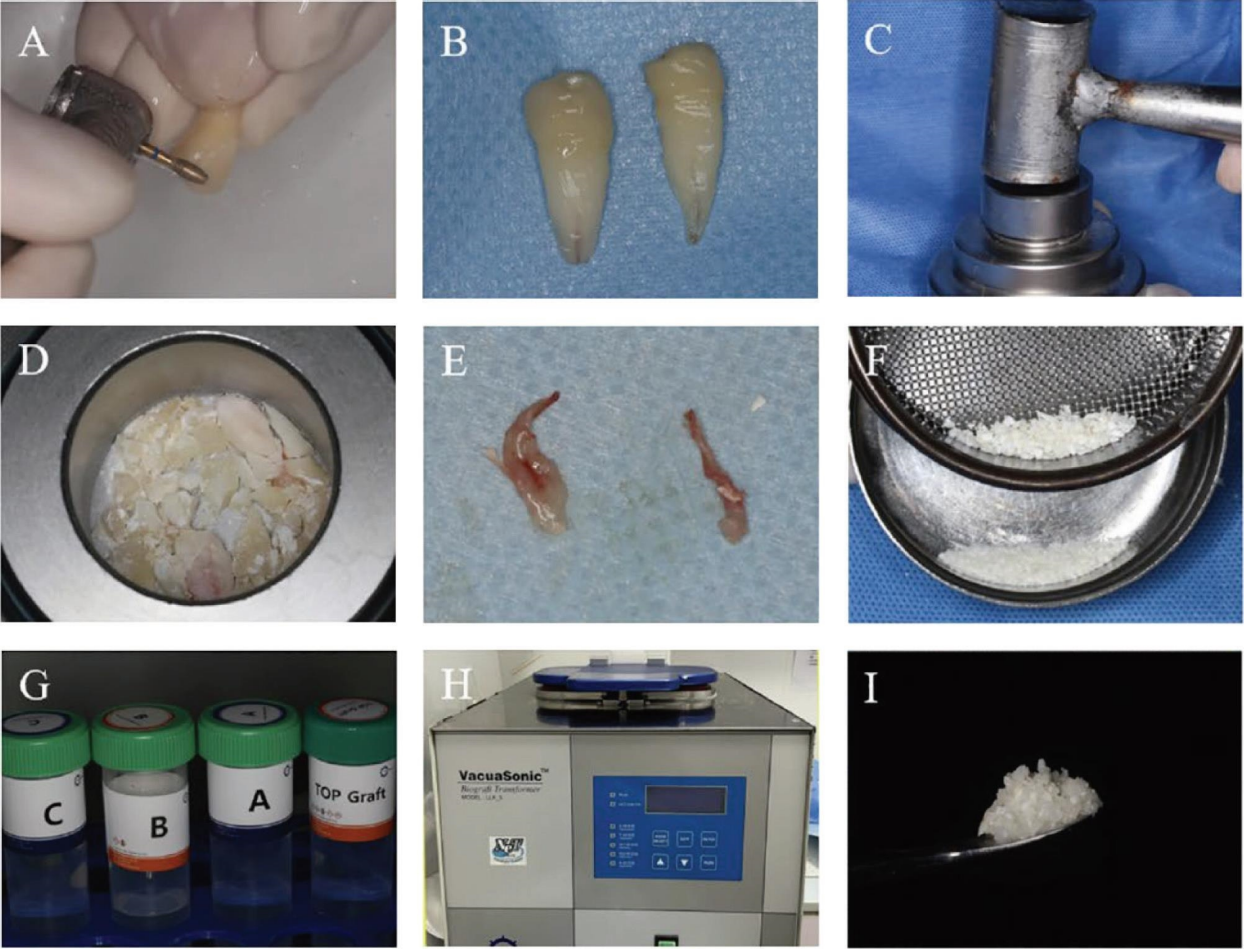
Vacuasonic visual representation of the particles graft preparation protocol (source: Xu et al. [32] demineralized dentin matrix promotes gingival healing in alveolar ridge preservation of premolars extracted for orthodontic reason: a split-mouth study. Front. Endocrinol. 14:1281649. doi: 10.3389/fendo.2023.1281649). (A) After the tooth extraction, the enamel and cementum of teeth are removed using a high-speed handpiece. (B) Teeth after the removal of enamel and cementum. (C) Powder kit tool for grinding dental tissue using hammer. (D) Dental tissue after initial grinding. (E) Removed dental pulp tissue. (F) Filter the grinding tissue through a 1 mm sieve to obtain dentin particles. (G) The decalsi PDM reagent used for demineralization, washing and sterilization of dentin particles. (H) Using the vacuasonic system device for programed treatment of dentin particles. (I) Dentin graft particles obtained after 30 min processing.

**Figure 5:**
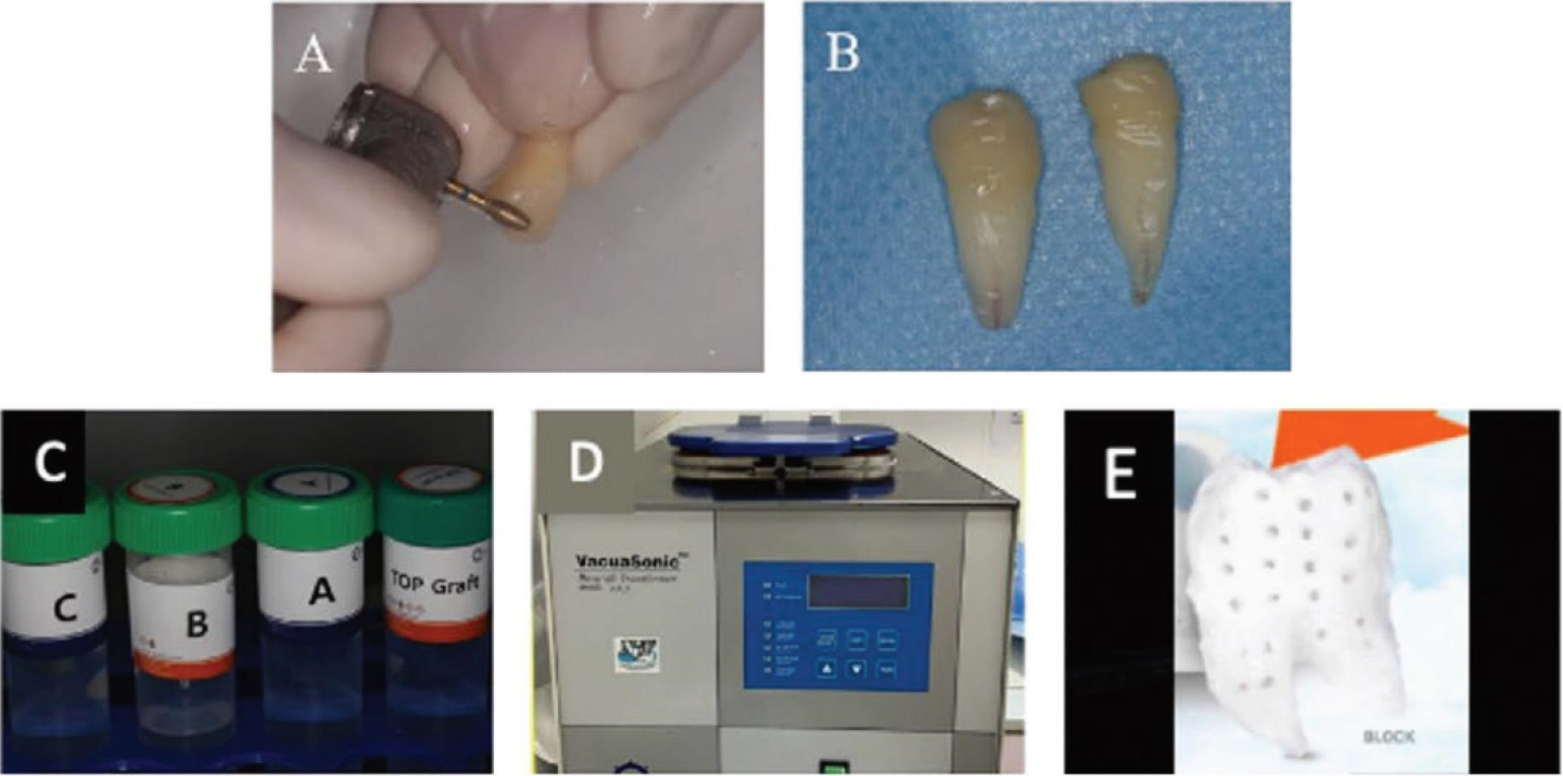
Vacuasonic visual representation of the block tooth graft preparation protocol (source: Xu et al. [32] demineralized dentin matrix promotes gingival healing in alveolar ridge preservation of premolars extracted for orthodontic reason: a split-mouth study. Front. Endocrinol. (A) After tooth extraction, the enamel and cementum of teeth are removed using a high-speed handpiece. (B) Teeth after the removal of enamel and cementum. (C) The decalsi DM reagent is used for demineralization, washing and sterilization of dentin particles. (D) Using the Vacuasonic system device for programed treatment of dentin particles. (E) Dm block obtained after 2 h processing.

It is evident that not all these procedures have been routinely applied, nor have all of them been incorporated into the functionality of a device for commercialization. In fact, to date, only four devices are available on the market for the use of teeth as grafting material. These are BonMaker (Figure 1), Tooth Transformer (Figure 2), Smart Dentin Grinder (Figure 3), and VacuaSonic (Figures 4 and 5). The extracted tooth treatment protocol of each device is summarized in Table 1.

All these devices have three stages, which are described as follows:

### 2.1. Step 1: Tooth Cleaning

After the extraction, the tooth must be cleaned of any residues of calculus, caries, soft tissues, and restorations using diamond burs or ultrasonic tips under abundant irrigation to avoid the possibility of temperature rise. The temperature should never exceed the threshold of 43°C to prevent the denaturation of autogenous proteins. Any filling materials must be eliminated, even cleaning in excess of the dental tissue on which the reconstruction lies, to avoid finding resins or other materials in the regeneration material. The prosthetic parts and cement must be cleaned in the same way.

Tooth Transformer [28], BonMaker, and VacuaSonic devices allow the use of teeth with root canal therapies, in which case, cleaning performed mechanically is preferred. BonMaker, VacuaSonic, and Smart Dentin Grinder devices, in which the sieve is used to separate the granules according to size, it is advisable to remove with tweezers the parts not congruent with the dental tissue after grinding.

Tooth Transformer, which requires the tooth to be sectioned for grinding, recommends cleaning the root canal treatment residues during the sectioning phase, thus allowing the cleaning of small sections to be more easily visible by optical magnification.

### 2.2. Step 2: Tooth Grinding

Two devices (BonMaker and VacuaSonic) involve the use of a hammer and pestle to crush the tooth. After crushing, the tooth is placed in a nonsterilizable high-speed mill. The granules are then separated by a manual sieve.

This sieve has two meshes and separates the fragments of different sizes. The larger ones, sized 850 μm, will remain blocked by the first filter, while the thinner ones, sized 450 μm, will pass through the second filter, ending up in the lower plate. Smart Dentin Grinder also uses a high-speed mill, which, however, differs from the previous ones as it is disposable and contains an automatic sieving and vibrating system. After grinding, a vibration system is activated which filters the tooth through the holes on the bottom of the grinder, blocking granules larger than 1200 μm, while another drawer sieve filters granules larger than 300 μm. The high-speed crushing ensures rapidity and determines the pulverization of part of the tooth. Minetti et al. [33] Tooth Transformer uses a multiuse sterilizable system, which works at low speed without loss of pulverized dental substance, but it has the disadvantage of not allowing the insertion of a whole tooth into the grinder, and therefore, the sample must be sectioned within dimensions before the insertion.

### 2.3. Step 3: Treatment by Device

#### 2.3.1. BonMaker

The tooth, crushed properly, must be inserted manually into a cylindrical container in plastic sterilized multipurpose material (Bonbin). The Bonbin containing the granulate must be inserted into a slot on the upper front part of the machine. The liquids, contained in disposable bottles, must be emptied manually into the respective cavities following a color code. A bottle to be tightened onto the upper part of the device must also be filled with physiological saline solution.

The device can treat even whole teeth, to be inserted into the Bonbin, which must previously be perforated with a specifically designed bur. Specific liquids are available, with the same procedure, for treating the block. At the end of the treatment, which lasts about 26 min, the material is extracted from the Bonbin. Exhausted and contaminated liquids are collected in a glass bottle situated behind a door in the front of the device, which must be emptied after a few uses. The composition of the liquids has not been clarified in any publication. In 2016, at the Polytechnic University of Milan, the liquids used were therefore subjected to an analysis, and the results were as follows: granular formulation: hydrochloric acid (HCl) 0.45 M-H_2_O_2_ 130 volumes-ethanol 62.6% chloroform 31.3% water 6.1% + washing saline solution; block formulation: HCl 0.56 M-H_2_O_2_ 120 volumes-ethanol 47.2% chloroform 47.2% water 5.6% + washing saline solution [31].

#### 2.3.2. Vacuasonic

The tooth, made granular, must be inserted manually into a disposable plastic phial. After screwing a cap containing the formulation, according to a sequence numbered from 1 to 3, the disposable phial with the tooth is inserted inside a grid, under a trap door on the upper part of the device. The device must be previously filled with an aqueous solution. The granulate must then be moved to phial no. 2 and no. 3, repeating the procedure. The device consists of a large ultrasonic tank that amplifies the action of the individual reagents. The same procedure should be followed with the whole tooth to obtain a block graft. The liquids used are: 0.6 M HCl + peracetic acid + ethanol + phosphate wash buffer solution [29, 30, 34].

#### 2.3.3. Smart Dentin Grinder

The granulated tooth from the mill is placed in a glass bowl, into which the first liquid contained in plastic bottles is added according to a color code.

After the time required for the procedure, the liquid is eliminated using a gauze or a pipette, and then the second liquid is introduced. Recently, the manufacturer presented a third liquid to be used if you want to have demineralization (EDTA). Liquids need to be added manually and removed manually using gauze or pipettes. It is also possible to dry the mixture with the intention of storing it on a hot plate at 140°C, manually placing the glass bowl over the hot plate. In literature, there seem to be different methods of use, all made possible by manual use of the device and varying the times on personal considerations. In 2014, Bindermann et al. [35] proposed to immerse the particulate matter for 10 min in a liquid called alcohol cleanser, consisting of 0.5 M NaOH and 30% ethyl alcohol, and then use it after rinsing twice with a phosphate-buffered saline (PBS) buffer solution. Alternatively, the still-wet particulate can be placed on a hot plate at 140°C for 5 min to have a bacteria-free particulate for a ready or later use. In 2016, Hallel et al. [36] started to immerse the obtained granules in 0.5 M NaOH and 20% ethyl alcohol for 10 min and then to eliminate the solution by means of a pipette or gauze and, finally, to rinse all with the PBS buffer solution for 3 min.

In 2017, other authors [19] indicated that the cleanser consists of 0.5 M NaOH and 20% ethyl alcohol to be used for 10 min, followed by PBS for 3 min, then eliminated with gauze.

Guirado et al. [20], 1 year later, indicated a 15-min soaking time, then a 5-min PBS wash. The same author [21] recommended using the cleanser for 10 min, then for 2 min in EDTA solution, and finally in saline for 3 min. In 2019, Cardaropoli et al. [37] presented the protocol of a 0.5% NaOH and 30% ethyl alcohol bath for 10 min.

More recently, Ozkahraman et al. [38] proposed the combined treatment of 20% ethanol + 80% sodium hypochlorite for 10 min. The solution was then absorbed and removed with a gauze pad. The dentinal particles were then kept in PBS solution for 3 min

In the same year, Kozuma et al. [39] published a new protocol in which the particulate dentin was immersed in basic alcohol cleanser in a sterile container for 7 min to dissolve all organic debris and bacteria. Then, the particulate was washed with sterile saline for 3 min.

Di Biase et al. [40], in 2020, proposed a treatment involving the immersion of dentin in 0.5M NaOH and 20% ethanol solution for 10 min, to dissolve all the organic remains from the tubules. The particulate was first dried with sterile gauze, then rinsed two times (3 min each) with phosphate-buffered saline solution to remove all the NaOH and the ethanol [35–38, 40–44].

#### 2.3.4. Tooth Transformer

After inserting the sectioned and cleaned tooth inside the grinder, it is closed and placed in the device. The disposable part contains a cartridge with liquids and a cylinder with a cup for collecting the granulate. Both are inserted into the device in their respective slots. The cartridge is activated by piercing, and then, once the door is closed and the button is pressed, the process starts. The procedure is completely automatic and repeats the same steps each time. The first phase of grinding at low speed causes the granules to fall into the collection basket. The six liquids present in the cartridge tank fall by gravity after the automatic perforation of the lower membrane of the cartridge and start the process. The granules are subjected to UVA rays and ultrasonic vibrations with temperature variations always below 43°C to avoid damage to the proteins. At the end of the process, the used and contaminated liquids remain inside the cylindrical container, which can be disposed of. The liquids used have not been indicated in the literature. In this case, the authors received, for the first time, the possibility to publish the liquids used, which are constituted by 0.1 M HCl, 10% hydrogen peroxide, and demineralized water as a wash [31].

### 2.4. Definition of Relevant Parameters and Classification

All available devices have some points in common, fragmentation, detoxification, demineralization, and some differences as type of fragmentation, type of detoxification, type of demineralization, and type of procedure (automatic or manual).

Some authors have compared and analyzed the histological and clinical differences of three different dentin matrix-based biomaterials obtained from different devices. The results indicate substantial differences in the histological characteristics and basic analysis of the materials produced by the three devices, even though they were clinically similar.

This obviously implies that there are clear differences between the products of each individual device.

Dlucik et al. [45], in 2023, indicated there are structural and chemical differences in the dentin granules produced from different devices. From this point, it is necessary to create a classification [46].

### 2.5. Demineralization

**Figure 6:**
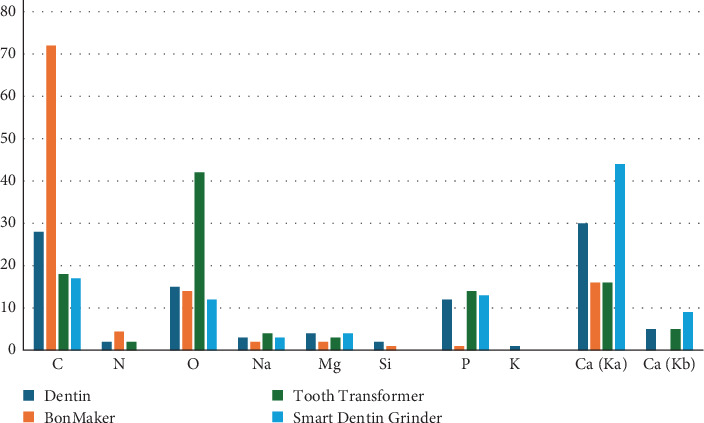
This table analyzes the values of the components of the dental-derived product originating from various devices from the Dlucik article [45].

Some authors have suggested complete demineralization. Dlucik et al. [45, 46], analyzing various devices, assessed the amount of Ca and P to evaluate the mineralization of the granulate after treatment. He found that BonMaker showed a significant reduction in minerals, confirming the more advanced demineralization achieved by the Korean device (Figure 6).

In BonMaker specimens, the major elements sampled were C and O, with only small traces of N, Mg, and Si, thus indicating a deep demineralization of the sample examined.

Tooth Transformer samples showed demineralization was not as effective as in the BonMaker and which indicates the partial demineralization.

In Smart Dentin Grinder specimens, the highest element sampled was Ca and which indicates less demineralization. Therefore, one distinguishing factor is the level of demineralization.

This allows the devices to be divided into three categories regarding demineralization.

Dlucik et al. [45, 46] studies analyzed the values of the components of the dental-derived product originating from various devices (Figure 6). The table indicates substantial differences for each preparation, suggesting a corresponding difference in clinical outcomes. Bono et al. [47] conducted a similar test on dentin, dentin treated with Tooth Transformer, and Bio-Oss. Here too, the results between the treated and the nontreated are different (Table 2).

Dlucik in the same articles as shown, in terms of composition of organic matter, BonMaker and Tooth Transformer samples showed a higher intensity in spectroscopy when compared to Smart Dentin Grinder.

Koga et al. [48] hypothesized that bone regeneration depends on the degree of tooth demineralization, and in his study analyzed different granule dimensions and different demineralization degrees, and the conclusion was that partially demineralized dentin matrix with larger particle size induced prominent bone regeneration.

In only one article, the presence of BMP-2 is demonstrated after a treatment using Tooth Transformer device by Franceschelli et al. [49]. No articles were found addressing this important aspect in relation to other devices.

### 2.6. Detoxification

Another key factor for successful tooth-derived bone regeneration is bacterial detoxification, probably the most important aspect in the regeneration field. We found that only one device has published an evaluation document on microbial contamination after the treatment with an assessment of bacterial load: the Tooth Transformer in Bono et al. [47] study. The fact that no publications about the other devices were found does not imply that tests on this important aspect have not been conducted [47].

### 2.7. Fragmentation

The third factor is the type of fragmentation used to create a granulate or, alternatively, the use of the whole tooth. Different shapes and surfaces are recognized during SEM analysis from Dlucik et al. [45, 46]. The importance of particle size is demonstrated by Koga et al. [48] studies. This is the reason why all the devices produce granule sizes between 0.4 to 1 mm. But there is a very important aspect, in fact, a literature review was conducted using the following keywords: “high-speed grinder” or “low-speed grinder” and “tooth.” The results for the last 10 years are as follows:

PubMed: 0 results, Web of Science (WOS): 50 results, and Scopus: 14 results. All the articles found are not relevant to dentistry.

The reasons for this could be attributed to the development of a new research field in regenerative dentistry that has gained momentum in recent years, focusing on the use of teeth as graft materials.

We have two different systems to grind the tooth: low speed and high speed. The differences were analyzed in 2024 with a sample of 40 human teeth [33, 50], and the result is that high speed need to use a sieve to recover the granules of the right dimensions. That implies a reduction of the volume produced. The low speed produces directly the granules of the right dimension which allows not to lose volume. That aspect is important because the use of autologous tooth is limited in the volume of the extracted tooth, of course. From this study, the percentage of the mean tooth lost with this high-speed grinder is 53.50% ± 9.89% of the tooth load. The low-speed grinder does not pulverize the dentin and creates a regular dimension of the tooth granules. The percentage of teeth loss is 9.16% ± 2.34%.

Some devices (Vacuasonic and BonMaker) allow the use of the entire tooth as a block. The limitation of this procedure is that the block is not replaced by bone but is incorporated, and obviously, it does not allow for implant placement. Therefore, the purpose of these procedures is primarily esthetic rehabilitation, not functional [51].

### 2.8. Manual or Automatic

The fourth factor is the use of either a manual or automated device. Another aspect worth discussing is the automation of tooth-derived graft preparation in the case of each device. Tooth Transformer is fully automated, while BonMaker, Smart Dentin Grinder, and Vacuasonic are semi-automated devices. The semi-automation of the procedure results in increased time spent by the surgeon in the process of preparation.

The reproducibility of a protocol through automation is the foundation of a scientific procedure. The lack of automation implies the possibility of variations in timing and liquid contact, leading to different reactions at the structural and surface levels of the granules at the end of the procedure. It is quite evident that this can result in high variability in outcomes, including negative results.

### 2.9. Concluding Remarks, Future Perspective, and Influence in Clinical Decision-Making

Based on all these considerations, we propose this initial classification of devices designed to produce graft material of dental origin (Table 3).

Demineralization can be total, partial, or absent, and the same applies to detoxification. Fragmentation can occur at high speed, low speed, or be absent, and the device can be either automatic or manual. The initials of the various processes used by each device form the basis of an acronym that encapsulates the key information for distinguishing one device from another.

Numerous clinical and in vitro studies highlight the qualities of teeth as graft material application and for each specific purpose [52, 53]. But the differences between one preparation and another require optimizing demineralization protocols, material characterization, consistency development, and handling techniques to ensure standardization or the recognition of these differences.

Further research is needed to identify the most appropriate demineralization conditions and particle sizes for clinical use in implant dentistry.

This classification may aid in the selection of the optimal device for practitioners seeking to utilize the tooth as graft material. Automation is the essential component for ensuring reproducible processing across all scientific fields. Therefore, system design should consistently prioritize minimizing the number of variables that could introduce unpredictable alterations. Ideally, future developments should aim toward a fully automated device, including the cleaning phases of the dental element

## Figures and Tables

**Table 1:** In the last 50 years scientists tried to discover the ideal procedure that could combine detoxification with partial demineralization, without damaging autogenous proteins and growth factors.

Tooth treatment procedures	Literature
Cleaned in 4% hydrogen peroxide, 70% ethanol for 10 min, stored at −20° C, reduced to 800–1000 µm powder, demineralized in 0.6 M HCl for 10 min, then washed in saline solution and sterilized with a peracetic acid–ethanol solution and then washed in saline	Park M, Mah YJ, Kim DH, et al. Demineralized deciduous tooth as a source of bone graft material: its biological and physicochemical characteristics. Oral Surg Oral Med Oral Pathol Oral Radiol 2015; 120:307–14

Demineralized in 0.6 m HCl for 5 days and then removed with prolonged washing in sterile 0.15 M NaCl	Yeomans JD, Urist MR. Bone induction by decalcified dentin implanted into oral, osseous, and muscle tissues. Arch Oral Biol 1967; 12:999–1008

Descaled in 0.6 M HCl for 72 h at 4°C, then immersed in many 70% alcohol baths, and rinsed in distilled water	Urist MR. Bone formation by autoinduction. Science 1965; 150 (3698):893–9

Demineralized by 0.5 M HCl at room temperature, created a powder treated with ethyl alcohol and water saturated with phenol for 30 min. Then rinsed in 70% ethanol and water, frozen in liquid nitrogen dehydrated with ethanol and ether overnight at 37°C	Huggins C, Wiseman S, Reddi AH. Transformation of Fibroblasts by allogeneic and xenogeneic transplants of demineralized tooth and bone. J Exp Med 1970; 132:1250–8

HCl 0.6 M	Nade N. Bone graft surgery reappraised: the contribution of the cell to ultimate success. Brit J Surg 1970; 57:752–6

EDTA	Nade N. Bone graft surgery reappraised: the contribution of the cell to ultimate success. Brit J Surg 1970; 57:752–6

Different HCl solutions: 48 h at 0.2 M, 48 h at 0.4 M, 48 h at 0,8 M, 48 h at 1 M, and 48 h at 2 M	Bang G. Induction of heterotopic bone formation by demineralized dentin: an experimental model in Guinea pigs. Scand J Dent Res 1973; 81:240–50

Demineralization by HNO_3_ (nitric acid)	Koga T, Minamizato T, Kawai Y, et al. Bone regeneration using dentin matrix depends on the degree of demineralization and particle size. PLoS One 2016; 11 (1): e0147235

Dimensions 500 µm in ice + B-TCB	Nampo T, Watahiki J, Enomoto A, et al. A new method for alveolar bone repair using extracted teeth for the graft material. J Periodontol - 81:1264–72

70% ethyl alcohol and then to Korea Tooth Bank	Kim KW. Bone Induction by demineralized dentin matrix in nude mouse muscles. Maxillofac Plast Reconstr Surg 2014; 36 (2):50–6

Crushed with high-speed Kometabio-alcohol to remove bacteria and physiological solution to remove alcohol 52	Calvo-Guirado JL, Cegarra del Pino P, Sapoznikov L, et al. A new procedure for processing extracted teeth for immediate grafting in postextraction sockets. An experimental study in American Fox Hound dogs. Ann Anat 2018; 217:14–23

Immersed in 70% ethyl alcohol and sent to Korea Tooth Bank, where they dehydrated with ethyl alcohol and a solution of ethyl ether, lyophilized, and disinfected with oxide of ethylene and packaged, and shipped to the surgeon	Kim SK, Kim SW, Kim KW. Effect on bone formation of the autogenous tooth graft in the treatment of peri-implant vertical bone defects in the minipigs. Maxillofac Plast Reconstr Surg 2015; 37:2

10% EDTA calcium hydroxide MTA for 14 days	Tomson PL, Grover LM, Lumley PJ, et al. Dissolution of bio-active dentin matrix components by mineral trioxide aggregate. J Dent 2007; 35:636–42

—	Graham L, Cooper PR, Cassidy N, et al. The effect of calcium hydroxide on solubilization of bio-active dentin matrix components. Biomaterials 2006; 27 (14):2865–73

Stored in sodium chloride solution, then immersed in ethanol for 20 h, pulp removed, minced in ice immersion, 10% EDTA, and 5 M PMSF for 14 days	Kim HS, Lee DS, Lee JH, et al. The effect of odontoblast conditioned media and dentin noncollagenous proteins on the differentiation and mineralization of cementoblasts in vitro. Arch Oral Biol 2009; 54:71–9

37% phosphoric acid for 15 s 3 weeks in 0.5 M EDTA at 4°C	Vennat E, Bogicevic C, Fleureau J-M, et al. Demineralized dentin 3D porosity and pore size distribution using mercury porosimetry. Dent Mater 2009; 25:729–35

Placed at −80° for 24 h 0.6 M HCl 1 week + chloroform and methanol for 24 h and then granulated with a Spex Industries shredder	Yagihashi K, Miyazawa K, Togari K, et al. Demineralized dentin matrix acts as a scaffold for repair of articular cartilage defects. Calcif Tissue Int 2009; 84:210–20

17% EDTA pH 7.5 15 min 17% EDTA pH 9 15 min 10% EDTA pH 7.5 15 min 10% EDTA pH 9 15 min	Parmar G, Chhatariya A. Demineralizing effect of EDTA at different concentration and pH-A spectrophotometer study. Endodont 2004; 16:54–7

Boiling in water for 2 h, 2 h in isopropranol, and dried at 100° and sterilized with gamma rays	Moharamzadeh K, Freeman C, Blackwood K. Processed bovine dentin as a bone substitute. Br J Oral Maxillofac Surg 2008; 46:110–3

Boiling water 30 min, 735°C calcination, and sintering at 1150° C for 1 h	Elkayar A, Elshazly Y, Assaad M. Properties of hydroxyapatite from bovine teeth. Bone Tissue Regen Insights 2009; 2:31–6

3 s of grinding, alcohol 0.5 M NaOH (sodium hydroxide) + 30% alcohol (alcohol solution) for 10 min rinse in saline solution	Cardaropoli D, Nevins M, Schupbach P. New bone formation using an extracted tooth as a biomaterial: a case report with histologic evidence. Int J Periodontics Restorative Dent 2019; 39 (2):156–63

0.6 m HCl at 2°C + 70% ethyl alcohol and gentamicin	Pinheiro Carvalho VA, de Oliveira Tosello D, de Castillo Salgado MA, et al. Histomorphometric analysis of homogenous demineralized dentin matrix as osteopromotive material in rabbit mandibles. Int J Oral Maxillofac Implants 2004; 19:679–86

	Gomes MF, Banzi EC, Destro MF, et al. Homogenous demineralized dentin matrix for application in cranioplasty of rabbits with alloxan-induced diabetes: histomorphometric analysis. Int J Oral Maxillofac Implants 2007; 22:939–47

	Gomes MF, Destro MF, Banzi EC, et al. Optical density of bone repair after implantation of homogenous demineralized dentin matrix in diabetic rabbits. Braz Oral Res 2008; 22:275–80

Liquid nitrogen −169°C for 2 weeks then in 70% ethyl alcohol 30–60 min	Al-Namnam NM, Shanmuhasuntharam P, Ha KO, et al. Processed allogenic dentin as a scaffold for bone healing: an in vivo study. Aust J Basic Appl Sci - 4:5932–40

10 EDTA at 25°C for 3 months	Reis-Filho CR, da Silva ER, Martins AB, et al. Demineralized human dentin matrix stimulates the expression of VEGF and accelerates the bone repair in tooth sockets of rats. Arch Oral Biol 2012; 57:469–76

10% EDTA for 3 min	de Oliveira GS, Miziara MN, Silva ER, et al. Enhanced bone formation during healing process of tooth sockets filled with demineralized human dentin matrix. Aust Dent J 2013; 58:326–32

Cleansed 10% H_2_O_2_ + pulverized + decalcified 1 group 2% H_2_SO_4_ 20 min/2 group 2% HCl 20 min/3 group 2% HNO_3_ 20 min/4 group 2% EDTA 20 min All rinsed with physiological solution 3 times 10 min and disinfected with ethylene oxide	Jang HS, Kim SG, Lim SC, et al. Osteogenic ability according to the decalcified modality of auto-tooth bone grafts in peri-implant defects in dogs. Implant Dent 2014; 23:482–8

Washed 10% H_2_O_2_, dehydrated ethyl alcohol, degreased ether ethyl-freeze-dried and disinfected with ethylene oxide	Kim SK, Kim SW, Kim KW. Effect on bone formation of the autogenous tooth graft in the treatment of peri-implant vertical bone defects in the minipigs. Maxillofac Plast Reconstr Surg 2015; 37:2

In ultrasonic tank with 75% alcohol then sintered at 800°C and gamma rays	Huang YC, Lew WZ, Feng SW, et al. Histomorphometric and transcriptome evaluation of early healing bone treated with a novel human particulate dentin powder. Biomed Mater 2016; 12:015004

0.2 HCl for 144 h, lyophilized, and sterilized with ethyl alcohol	Movin S, Borring-Møller G. Regeneration of infrabony periodontal defects in humans after implantation of allogenic demineralized dentin. J Clin Periodontol 1982; 9:141–7

Chopped with a coffee grinder >700 rpm. Demineralized with 1 M lactic acid for 15–20 min, rinsed with saline for 60 s	Joshi CP, Dani NH, Khedkar SU. Alveolar ridge preservation using autogenous tooth graft versus beta-tricalcium phosphate alloplast: a randomized, controlled, prospective, and clinical pilot study. J Indian Soc Periodontol 2016; 20:429–34

Immersed in a gentamicin solution + 70% ethyl alcohol at 2°C	Gomes MF, de Abreu PP, Morosolli AR, et al. Densitometric analysis of the autogenous demineralized dentin matrix on the dental socket wound healing process in humans. Braz Oral Res 2006; 20:324–30

Calcium chloride	Urist MR. Bone formation by autoinduction. Science 1965; 150:893–9

EDTA + formic acid + citric acid	Urist MR. Bone formation by autoinduction. Science 1965; 150:893–9

Lactic acid	Urist MR. Bone formation by autoinduction. Science 1965; 150:893–9

Heat 70°C	Urist MR. Bone formation by autoinduction. Science 1965; 150:893–9

Nitric acid + HCl + β-propiolactone	Urist MR. Bone formation by autoinduction. Science 1965; 150:893–9

DNFB (dinitrofluorobenzene)	Urist MR. Bone formation by autoinduction. Science 1965; 150:893–9

IAA (iodoacetamide) + HCl	Urist MR. Bone formation by autoinduction. Science 1965; 150:893–9

Tooth treatment protocols of the only four devices available in the market	

BonMaker (Figure 1) (simple formulation) HCl 0.45 M-H_2_O_2_ 130 volumes-ethanol 62.6% chloroform 31.3% water 6.1%; (aggressive formulation) HCl 0.56 M-H_2_O_2_ 120 volumes-ethanol 47.2% chloroform 47.2% water 5.6%	Tests performed at University of Milan (2006)

Tooth Transformer (Figure 2) 0.1 M HCl + H_2_O_2_ 10% (34 volumes)	Minetti E, Casasco A, Casasco M, Corbella S, Giacometti, E, Ho HKL, Palermo A, Savadori P, Taschieri S. Bone regeneration in implantology: tooth as a graft. 2021 EDRA ed. ISBN: 978-88-214-5353

Crushed for 3 s with Smart Dentin Grinder 300–1200 µm (Figure 3) 10 min in 0.5 M NaOH + 30% alcohol (called basic alcohol) rinsed twice with saline sulfate solution. If necessary, store for the future, place at 140°C for 5 min	Bindermann I, Hallel G, Nardy C, et al. A novel procedure to process extracted teeth for immediate grafting of autogenous dentin. J Interdiscipl Med Dent Sci 2014; 2:154

VacuaSonic (Figures 4 and 5) Teeth were demineralized in a 0.6 N hydrochloride and 10 min of sterilization using sterilization reagents (peracetic acid–ethanol solution). The demineralized particulate tooth bone was washed and neutralized using phosphatebuffered saline	Lee E-Y, Kim E-S, Kim K-W. Scanning electron microscopy and energy dispersive X-ray spectroscopy studies on processed tooth graft material by vacuum-ultrasonic acceleration. Maxillofac Plast Reconstr Surg 2014; 36(3):103−10 [29] Tulio AV, Kang CD, Fabian OA, et al. Socket preservation using demineralized tooth graft: a case series report with histological analysis. Int J Growth Factors Stem Cells Dent 2020; 3:27–34 [30]

*Note:* This table lists all the chemical/physical procedures proposed over the years to treat the autogenous teeth to transform them into suitable grafting material [31].

**Table 2:** Bono et al. [47] conducted this test with the dentin treated with Tooth Transformer and the results between non treated dentin and treated dentin are different.

Sample	C (%)	O (%)	P (%)	Ca (%)
Dentin	24.02	4.98	8.59	16.56
Demineralized dentin	60.02	26.06	5.04	8.59
Enamel	12.90	55.21	11.04	20.25
Demineralized enamel	16.13	58.26	9.99	15.02
Bio-Oss	15.95	62.32	8.62	12.45

**Table 3:** Possible autogenous tooth graft derived classification basis on transforming devices.

1-Demineralization	2-Detoxification	3-Fragmentation	4-Manual or automatic
Totally	Totally	Low speed	Manual
Partially	Partially	Hight speed	Automatic
None	None	None	—

Device		Acronym P = partially T = totally N = none L = low H = hight A = automatic M = manual	

Tooth Transformer		PTLA	
Smart Dentin Grinder		NPHM	
Vacuasonic particles graft		TTHM	
Vacuasonic block		TTNM	
BonMaker particles graft		TPHM	
BonMaker block		TPNM	

## Data Availability

All data and materials are available from Elio Minetti in Milan, Italy.
