# Correction to “New Classification of Autologous Tooth‐Derived Grafting Materials: Fundamental Concepts”

**DOI:** 10.1155/ijod/9826837

**Published:** 2025-12-30

E. Minetti, S. Taschieri, M. Berardini, and S. Corbella, “New Classification of Autologous Tooth‐Derived Grafting Materials: Fundamental Concepts,” *International Journal of Dentistry* 2025 (2025): 6646405, https://doi.org/10.1155/ijod/6646405

In the article titled “New Classification of Autologous Tooth‐Derived Grafting Materials: Fundamental Concepts,” the affiliation number 3 was included by error. The correct affiliation list and author affiliations are shown below:

Elio Minetti,^1^ Silvio Taschieri,^1,2^ Marco Berardini,^3^ and Stefano Corbella,^1,2^

^1^Department of Biomedical, Surgical, and Dental Science, University of Milan, Milan, Italy

^2^IRCCS Orthopedic Institute “Galeazzi,” Milan, Italy

^3^Private Practitioner, Pescara, Italy

We apologize for this error.

